# Gene expression to mitochondrial metabolism: Variability among cultured *Trypanosoma cruzi* strains

**DOI:** 10.1371/journal.pone.0197983

**Published:** 2018-05-30

**Authors:** Murat C. Kalem, Evgeny S. Gerasimov, Pamela K. Vu, Sara L. Zimmer

**Affiliations:** 1 Department of Biology, University of Minnesota Duluth, Duluth, Minnesota, United States of America; 2 Department of Biomedical Sciences, University of Minnesota Medical School, Duluth campus, Duluth, Minnesota, United States of America; 3 Faculty of Biology, M.V. Lomonosov Moscow State University, Moscow, Russia; University of Ostrava, CZECH REPUBLIC

## Abstract

The insect-transmitted protozoan parasite *Trypanosoma cruzi* experiences changes in nutrient availability and rate of flux through different metabolic pathways across its life cycle. The species encompasses much genetic diversity of both the nuclear and mitochondrial genomes among isolated strains. The genetic or expression variation of both genomes are likely to impact metabolic responses to environmental stimuli, and even steady state metabolic function, among strains. To begin formal characterization these differences, we compared aspects of metabolism between genetically similar strains CL Brener and Tulahuen with less similar Esmeraldo and Sylvio X10 strains in a culture environment. Epimastigotes of all strains took up glucose at similar rates. However, the degree of medium acidification that could be observed when glucose was absent from the medium varied by strain, indicating potential differences in excreted metabolic byproducts. Our main focus was differences related to electron transport chain function. We observed differences in ATP-coupled respiration and maximal respiratory capacity, mitochondrial membrane potential, and mitochondrial morphology between strains, despite the fact that abundances of two nuclear-encoded proteins of the electron transport chain are similar between strains. RNA sequencing reveals strain-specific differences in abundances of mRNAs encoding proteins of the respiratory chain but also other metabolic processes. From these differences in metabolism and mitochondrial phenotypes we have generated tentative models for the differential metabolic fluxes or differences in gene expression that may underlie these results.

## Introduction

Fundamental biochemical and gene expression pathways of the disease-causing, insect transmitted trypanosomatids are often deciphered in the model organism *Trypanosoma brucei*. Gene expression and flux through major metabolic pathways are known to dramatically differ between *T*. *brucei* proliferative insect and mammalian life stages [1–4]. However, other pathogenic trypanosomatids differ from *T*. *brucei* in important aspects of their life cycle. *Trypanosoma cruzi*, causative agent of Chagas disease, undergoes starvation, pH changes, and passage through the entire insect digestive tract in its transition to its mammalian infective stage [5], a transition very different than that of *T*. *brucei*. Furthermore, once in the mammalian host, *T*. *cruzi* replicates intracellularly rather than in the bloodstream as does *T*. *brucei* [1,6]. Utilization of metabolic pathways, particularly across the pathogen life cycle, must therefore be studied for each trypanosomatid individually.

Expression of the trypanosomatid mitochondrial genome, which encodes primarily over a dozen mRNAs of the electron transport chain (ETC) utilized in oxidative phosphorylation, has been extensively studied in *T*. *brucei* (reviewed in [7–11]). In *T*. *cruzi*, its regulation across the life cycle remains poorly understood. We have reported that during *in vitro* transition from the replicative to the infectious insect form, and also when deprived of all nutrient sources, mature mRNAs of the mitochondrial genome increase in abundance [12]. However, we were not able to determine how this change of the mitochondrial transcriptome functionally relates to *T*. *cruzi* metabolism. Our long-term goal is to determine how environmentally- or developmentally-stimulated changes in mitochondrial gene expression are played out at the functional level, particularly their impact on oxidative phosphorylation and other functions of the ETC and its components. However, products of the mitochondrial genome associate with products of the nuclear genome, so nuclear genetic diversity of *T*. *cruzi* strains is also an important consideration in pursuing this goal.

The species *T*. *cruzi* encompasses much genetic diversity among many characterized strains [13]. Strains of geographically and genetically distinct *T*. *cruzi* are currently organized into discrete typing units, or DTUs [14]. Different DTUs have been correlated with different Chagas disease outcomes, host species, and geographical ranges [13–15], and differ in size of their nuclear genome [16,17]. Furthermore, the evolution of extant *T*. *cruzi* strains includes events resulting in hybrid nuclear genomes of strains in two DTUs [18]. Our studies on *T*. *cruzi* mitochondrial gene expression [12] have focused on the hybrid strain CL Brener of DTU VI [19]. The *T*. *cruzi* CL Brener maxicircle (the mRNA- and rRNA-encoding portion of the mitochondrial genome) was sequenced in conjunction with the maxicircles of two strains from other DTUs, Sylvio X10 of DTU I, and Esmeraldo of DTU II [20–22]. When compared, their maxicircles exhibited frequent substitutions and indels in the expressed portion, although most indels would likely be resolved by a unique trypanosome mitochondrial RNA editing phenomenon [22]. Along with both nuclear and mitochondrial genetic diversity, the potential that differences in RNA expression levels of individual genes could vary between strains. This possibility has yet to be investigated.

Here, we elucidate the extent of *T*. *cruzi* strain-specific metabolic (mainly mitochondrial) variability and nuclear gene expression variability in strains that are attractive subjects of *in vitro* molecular studies of either the mitochondrial or nuclear genomes. While cell culture studies addressing strain differences in mitochondrial metabolism can be found [23,24], other comparisons have focused on glycolysis and cell characteristics peripherally related to mitochondrial function [25–29]. Furthermore, older studies often utilized strains that are not most commonly used in the laboratory today, or for which we possess fully sequenced genomes. This study complements an in-progress investigation of strain-specific differences in expression of the *T*. *cruzi* mitochondrial genome. Knowledge gained could also have an impact on our understanding of *T*. *cruzi* pathogenesis and life cycle transitions. We performed our work in insect stage *T*. *cruzi* axenic culture, paying the most attention to parameters related to ETC function. To the three strains described above we added DTU VI strain Tulahuen, rationalizing that if strain differences were observed, CL Brener and Tulahuen would be expected to have similar phenotypes, as they are of the same DTU. The examined strains were found to be remarkably similar in terms of their culture glucose consumption, but this is nearly the only parameter consistent between strains. The strains acidify their culture medium at different rates, suggesting pathways in which their metabolism may differ. We identified differences in aspects of ETC function, mitochondrial membrane potential, and mitochondrial morphology between strains. Finally, we used high-throughput sequencing analysis of mRNA levels to reveal strain-specific differences in mRNAs of multiple metabolic pathways.

## Materials and methods

### Culture

Replicating insect stage (epimastigote) *T*. *cruzi* cells from strains CL Brener (original non-clonal strain utilized in *T*. *cruzi* genome project, acquired from laboratory of Roberto Docampo, University of Georgia), Tulahuen cl98 (ATCC^**®**^ 50829), Sylvio X10 (ATCC^**®**^ 50823) and Esmeraldo cl3 (ATCC^**®**^ 50794) were cultured in Liver Infusion Tryptose (LIT) medium [30] supplemented with 10% FBS and 20 μg/ml hemin (Sigma H9039) in 27°C, 5% CO_2_ incubators. The continually propagated source cells for all experiments were diluted 1:10 in supplemented LIT every 2–3 days to maintain the growth in exponential phase [12,31]. LIT growth studies were initiated with 0.5 x 10^6^ cells/ml of exponentially growing cells and every 2 days counts were performed with a hemocytometer. To measure growth in RPMI, parasites grown in LIT medium were collected and resuspended in 1 ml of fresh LIT (in order to exactly normalize starting conditions between replicates), then transferred to a flask containing 10 mL RPMI. Growth was measured in the same manner as in LIT, except that the starting concentration of the culture was 4.5 x 10^6^ cells/ml, which is the concentration required in order to obtain sufficient cells for downstream applications when this culture is used for *in vitro* transition from epimastigote to trypomastigote forms [12]. All other experiments utilized cultures initiated in the same way as the growth experiments, with cells harvested at 24 hours for cells grown in LIT (regular growth medium), and 4 days for cells grown in RPMI (restricted medium), unless otherwise noted.

### Glucose assay and pH analysis

Glucose concentrations in RPMI culture medium were determined as described previously [12], with filtered culture medium frozen upon collection and samples analyzed together at the end of the experiment. Culture pH of the same samples was determined by pH meter measurement (Fisher Scientific Benchtop Meter, AB150).

### Metabolic flux analysis: Mitochondrial bioenergetics profile

Oxygen consumption rates (OCR) and other parameters related to mitochondrial respiration of all strains were measured using a SeaHorse XF^e^96 extracellular flux analyzer (Agilent) as described previously by another group [32,33]. Briefly, 96-well assay plates were coated with 181.5 ng/μl CellTak (Corning) in 100 mM sodium bicarbonate pH 8 for at least 30 minutes. Wells were washed 3 times with Krebs-Henseleit Buffer (KHB) prior to plating 8 x 10^5^ cells/well of exponentially growing *T*. *cruzi* in LIT or *T*. *cruzi* grown 4 days in the RPMI culture that were resuspended in described supplemented XF medium, and handled as described [32,33].

The SeaHorse Mito Stress Kit (Agilent, 10315–100) was utilized as described [33]. Briefly, ETC inhibitors oligomycin (2.5 μM), FCCP (3μM), antimycin A (1μM) and rotenone (1 μM), (concentrations previously optimized for *T*. *cruzi* where typical respirometry profiles for epimastigotes and other stages can be found; [33]), were added as directed to sequentially inhibit the function of specific ETC complexes, and the resulting changes in oxygen utilization were captured by the flux analyzer during the experiment. Results were normalized to control for number of cells in each well contributing to per well consumption of oxygen measured, using SYBR Green I fluorescent dye (Invitrogen S77563; 10,000X) staining of the amount of cellular DNA in each well. This method of controlling for cell plating variability has been used previously for *Plasmodium* [34]. At assay completion, the 96-well plate was centrifuged and medium was removed from each well to be replaced by 100 μl 10X SYBR Green I in lysis solution (50 mM HEPES, 125 mM KCl, 12 mM MgCl_2_ and 1.6% (v/v) Triton X-100, pH 7.4). Following overnight incubation, fluorescence was detected (SpectraMax M3 plate reader). Fluorescence values were directly used to normalize the OCR data to the per well cell numbers with the normalization function in Wave Desktop version 2.3 (Agilent).

### Mitochondrial membrane potential

Parasites from exponentially growing LIT or from 4 days post-initiation restricted growth (RPMI) cultures were collected and stained with 1 μM tetramethylrhodamine methyl ester percholarate (TMRM) for 45 minutes at 27°C (as performed in [35]). Analysis at a 60 nM concentration of TMRM revealed a similar trend as with assay performed at the 1 μM concentration that we present here. Parasites were then transferred to 15 ml conical tubes and kept on ice until analysis on SONY SH800 Flow Cytometer. 100,000 events were analyzed per sample. Parasites treated with 100 μM FCCP (protonophore) were analyzed as a control for mitochondrial depolarization. Median fluorescent intensity (MFI) for each sample was calculated with FlowJo software (version 10.4). MFI of cells of each strain was presented relative to MFI of CL Brener in regular growth medium (set to 100%).

### Immunoblotting: Nuclear-encoded ETC complex subunits

One nuclear encoded subunit of ETC complex IV and one of ATP synthase were immunoblotted as described previously [12]. Cell lysates were obtained from exponentially growing LIT cultures and day 4 restricted growth cultures by lysing cells directly in 1X SDS-PAGE loading buffer. *Leishmania major* COXIV (LmjF.12.0670; complex IV, serum produced by Emmanuel Handman, Walter and Eliza Hall Institute of Medical Research emeritus) and *T*. *brucei* ATP synthase beta subunit ([36]; Tb927.3.1380) polyclonal antibodies were used to probe the *T*. *cruzi* homologue. Specificity of these antibodies to the *T*. *cruzi* homologue was confirmed previously [12]. Monoclonal anti-α-tubulin antibody (Sigma-Aldrich T5168) was used to detect α-tubulin as a loading control [37]. Li-COR IRDye 800CW goat anti-rabbit and Li-COR IRDye 680RD goat anti-mouse antibodies were secondary antibodies for ETC subunit and α-tubulin immunoblots, respectively. Blots were visualized on the Li-COR Odyssey Fc imaging system and analyzed with Image Studio software. Briefly, pixel density value of the detected COXIV or ATP synthase beta subunit bands were normalized to α-tubulin band pixel density. Then expression of each protein in each strain was determined relative to CL Brener.

### High-throughput RNA sequencing and differential expression analysis

Identities of *T*. *brucei* genes classified into each examined functional category can be found in S1 Table. *T*. *cruzi* homologues of these genes were identified by bidirectional best blast hit between proteins of both species with blastp (NCBI-blast package, version 2.6.0+), e-value cut-off 1e-10 and BLOSUM52 matrix.

Reads from libraries used for differential expression analysis are the total RNA-derived Sylvio X10 reads described in Gerasimov et al. [38], and Esmeraldo and CL Brener strain reads obtained identically at the same time (part of the same dataset). Reads were trimmed for quality and of adaptor sequences using *Trimmomatic* version 0.36 [39]. As *T*. *cruzi* strains display high levels of gene synteny with insertions and deletions more prevalent in intergenic and repetitive regions, we used one genome reference to align all our datasets: the assembled nuclear genome of *Trypanosoma cruzi* CL Brener [40]. Two biological replicate read samples per strain were mapped using *STAR* version 2.4.0 [41], or *bwa* version 0.7.12 [42] in single-end mode (granting us computational replicates corresponding to read mates), allowing 2 mismatches per 100 nucleotides of read length. Only unique alignments were utilized for read counts. Read counts per CDS were obtained using the *BEDTools* package [43]. Differential expression analyses were done with two statistical methods. *Bwa*-generated read counts were further processed with *DEseq2* package version 1.18 [44] and *STAR*-generated read counts were processed with *EdgeR* version 3.20.2 [45]. Resulting differentially expressed gene lists were filtered by FDR < 0.001 and fold change > 3x. Filtered *EdgeR* output had fewer differentially expressed genes, but over 89% of those were also differentially expressed with similar fold change according to *DEseq*. The more conservative *EdgeR* results are those presented in the text and tables.

### Fluorescent microscopy: Mitochondrion morphology

Exponentially growing parasites in regular growth medium were collected and resuspended in 10 mL PBS-G (1X PBS, 0.1% glucose). Cells were transferred to flasks and MitoTracker Deep Far Red (ThermoFisher M22426) added at the final concentration of 250 nM. Cells were incubated for 20 minutes at 27°C, collected and fixed with paraformaldehyde and counter-stained with DAPI, and then slides were prepared as described previously [12]. Slides were visualized on a Carl Zeiss Laser Scanning Microscope 710 using 63x oil DIC objective and MitoTracker excited with the 637nm laser. DAPI was excited with the 405-diode laser. Attained images were analyzed using ZEN 2.3 lite software. Cells were qualitatively compared for the differences in mitochondrion morphology. Representative images from 3 biological replicates are presented.

### Statistical analysis

Graph Pad Prism version 6 was used to calculate means and SEMs, ANOVAs and *post hoc* Tukey’s HSD tests.

## Results

### Selected strains exhibit different culture growth profiles

**Fig 1:**
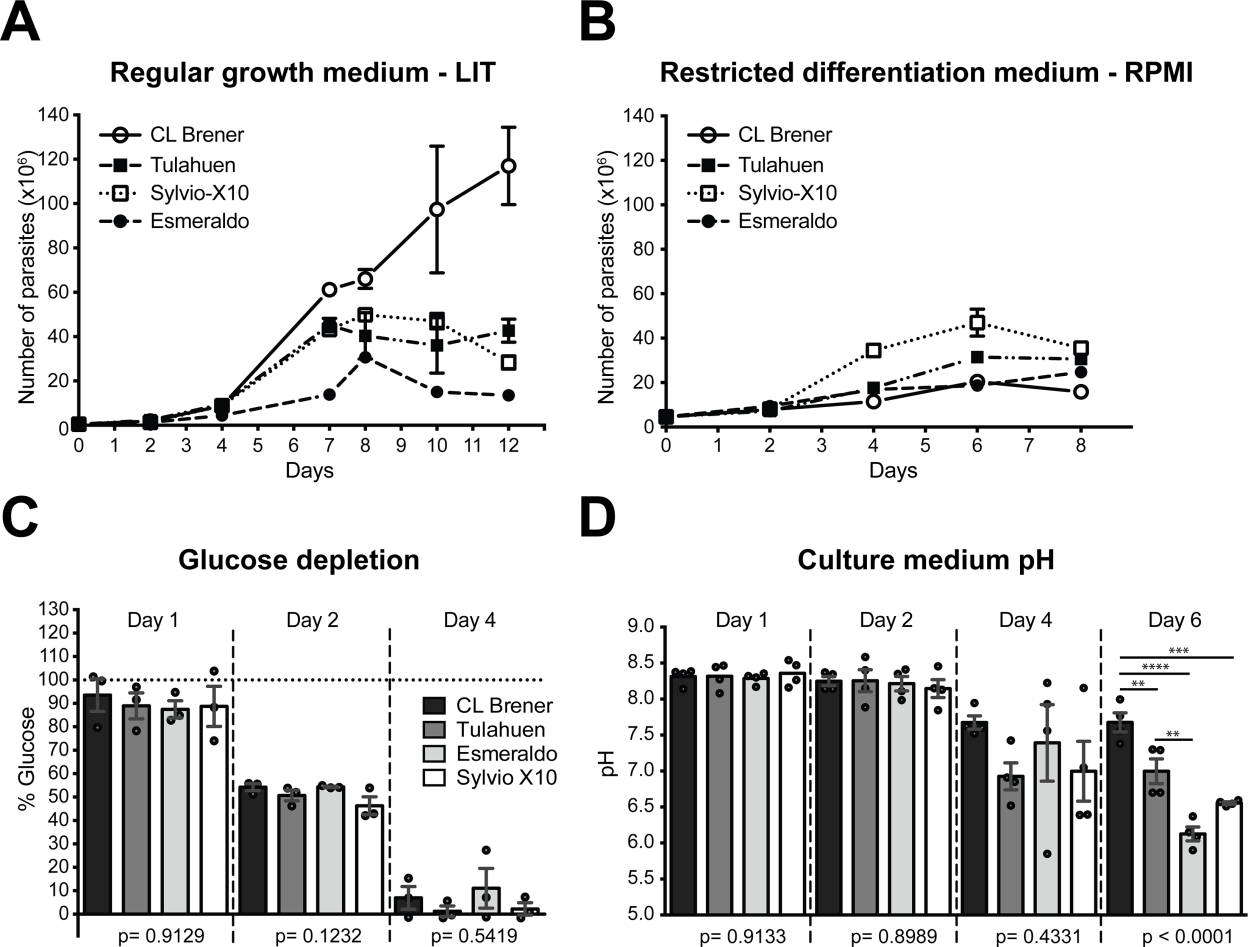
Growth of *Trypanosoma cruzi* strains and associated changes in medium glucose and pH levels. **A.** Growth of *T*. *cruzi* strains in regular growth medium, LIT. Cultures from 4 strains of *T*. *cruzi* with starting concentration of 0.5 x 10^6^ cells/ml were counted every 2 days by hemocytometer. **B.** Growth of *T*. *cruzi* strains in cultures started with 5 x 10^7^ cells resuspended in 1 ml of fresh, supplemented LIT medium added to a flask containing 10 ml RPMI (restricted medium). Each data point represents the mean of 3 replicates. Error bars represent the standard error of the mean (SEM). **C.** Medium glucose depletion. Cells from each strain were grown in restricted medium. Glucose concentrations were measured using a colorimetric assay at various time points. y-axis shows the percent culture glucose remaining relative to that of the initial culture. Horizontal dotted line represents level of glucose at the start of the experiment. Bars represent the means of 3 biological replicates; data points represent each biological replicate. Error bars show the SEM. Although analysis of some replicates was carried out up to 8 days, glucose was never detected beyond the day 4 time point shown. **D.** Extracellular acidification. Cells from each strain were grown in restricted medium. Culture medium was collected at various time points, centrifuged and filtered to be free of parasites, frozen for storage, and then later thawed and pH was measured using a standard pH meter. Bars represent the means of 3 biological replicates; data points represent each technical replicate. Error bars show the SEM. One-way analysis of variance (ANOVA) was used to identify the differences. *Post-hoc* Tukey’s test was performed when ANOVA revealed significant effects. ** represents p ≤ 0.01, *** represents p ≤ 0.001 and **** represents p ≤ 0.0001.

We first compared growth and life stage differentiation of all four strains as these have not previously been directly compared. Despite the fact that all have been extensively adapted to growth in culture, in normal rich medium during exponential growth, Esmeraldo grew more slowly than the three other strains that grew more similarly (days 1–7). However, strains’ stationary phase maximum densities varied within an ~ 10-fold range (days 9–10; Fig 1A), with CL Brener achieving the highest concentration and Esmeraldo the lowest. A more challenging restricted medium (RPMI) contains a defined, and thus narrower range of nutrient sources and other additives and was supplemented with very limited FBS compared to normal medium conditions. Strains grown in the restricted medium did not exhibit separate exponential and stationary growth phases. Instead they grew slowly throughout the 8-day experiment (Fig 1B). After 2 days in culture, Sylvio X10 grew most rapidly of all the strains. Interestingly, CL Brener, which grew best in normal medium, grew slowest and achieved the lowest cell concentration at the end of the experiment. However, cell concentrations at any measured day did not differ more than three-fold across all strains. These growth studies suggested that between these common laboratory strains, differing rates of flux through various metabolic pathways were likely, as has been observed in specific pathways between other strain comparisons (e.g. [23,24,28]).

We have previously used restricted medium conditions to analyze epimastigote to trypomastigote (infectious stage) transition in CL Brener *in vitro* [12]. We also now analyzed this for all four strains, since heterogeneity of response to other *in vitro* differentiation conditions between other strains has been reported e.g. [26,29], and we may eventually want to explore metabolism and gene expression in culture differentiated cells. We also saw differences in differentiation. Microscopic analysis of the restricted medium cultures after 8 days revealed that strain Esmeraldo did not undergo differentiation to become culture-induced trypomastigotes; however, CL Brener, Tulahuen and Sylvio X10 cultures respectively contained 31.4%, 43.2% and 42.4% cells that could be classified as either intermediate [12,46] or trypomastigote life stages (S1 Fig). In terms of life cycle differentiation by this method, strain Esmeraldo appears to be the major outlier.

### *T*. *cruzi s*trains deplete extracellular glucose at similar rates and acidify the culture medium at different rates

When trypanosomes are provided with multiple nutrient sources, glucose is known to be the preferred energy source [31,47–50]. We first wished to confirm that our strains did not exhibit obvious differences in glucose uptake or utilization. We tested rate of glucose depletion in our growth-restricted cultures where it is unlikely that large differentials in cell accumulation over the course of the assay would complicate analysis. We found that different strains depleted glucose from medium at nearly identical rates (Fig 1C), so at the very least, glucose uptake from the medium is consistent among the strains.

The fraction of imported glucose to fuel glycolysis and the pentose phosphate pathway (that diverge after the first enzymatic step in glycolysis) is known to vary between *T*. *cruzi strains* [28,51]. Furthermore, the amount of NADPH produced by that pathway is known to differ between strains CL Brener and Esmeraldo [23]. One thing that will influence these ratios is the amount of NADPH needed that, through a trypanosome-specific pathway, can counter the reactive oxygen species (ROS) generated by mitochondrial processes, including electron transport. Therefore, strain-specific differences in ETC function resulting in more or less ROS production could influence the degree to which the pentose phosphate pathways is utilized. Additionally, after the generation of phosphoenolpyruvate in glycolysis, the downstream products can be converted to known excreted metabolites alanine in the cytosol, excreted alanine and succinate in the glycosome (the trypanosomatid organelle in which the first six steps of glycolysis occur) or be further metabolized in the mitochondrion. Therefore, although glucose uptake is consistent across these strains, once glucose enters the glycosome to be catabolized, its utilization may ultimately differ.

During growth measurement, we noted strain-specific color variation in culture medium, reflecting differences in medium acidification, as the medium has a pH indicator. *T*. *cruzi* excretes acidic metabolic byproducts that would drop the culture pH. We therefore quantified acidification rates of each strain over time. During this analysis, cell death among strains was not appreciably different, as flow cytometric analysis of propidium iodide exclusion did not reveal signs of cell death. Extracellular acidification rates were strain specific starting from day 4, the same time point at which glucose in the medium was nearly or completely depleted (Fig 1D). As noted, differences in the ratio of phosphoenolpyruvate directed through various downstream pathways would theoretically result in differing excreted products, but generation of large amounts of phosphoenolpyruvate would take place only in the presence of available glucose. In our experiment, cells have primarily extracellular amino acids from the medium and stored protein and lipid energy sources [52] at the time points in which differences in medium pH are observed. In these conditions, a number of pathways and parameters may be differentially utilized or regulated among *T*. *cruzi* strains to result in a differential release of acidic byproducts. These include substrate-level phosphorylation of amino acids, oxidative phosphorylation, and the specific amino acids and/or lipids preferred as substrates [1,31,53].

To examine some of these possibilities in more depth, two culture conditions were selected for additional experiments, cells in exponential growth in the rich LIT medium at a time point at which glucose is still abundant (24 hours), and in the more restrictive RPMI growth medium at the time point where culture glucose has been exhausted. The former is not a perfect standard because epimastigotes in their normal environment do not often experience such idealized growth conditions. The latter is not perfect because the cells could be experiencing a stress that is a cause of the slow growth in this condition. Using both conditions improves the rigor of the study.

### The ETC is utilized at different levels and with varying efficiencies among strains

**Fig 2:**
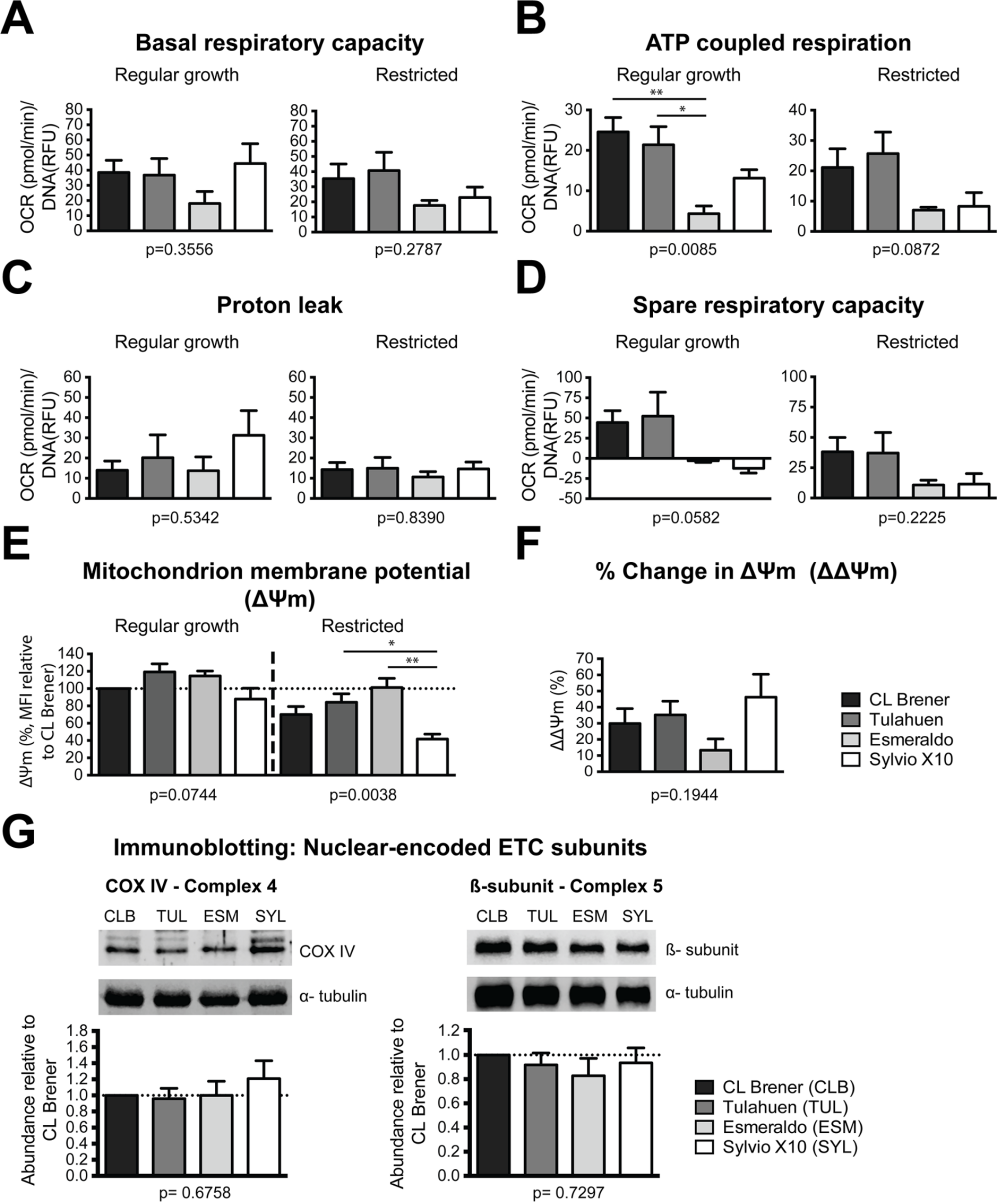
Mitochondrial electron transport chain (ETC) utilization and membrane potential in *Trypanosoma cruzi* strains. A mitochondrial stress test was used to determine ETC utilization among different strains in exponential growth in regular growth medium and after 4 days in restricted medium. Oxygen consumption rate measurements are normalized to SYBR Green 1 fluorescence. **A.** Basal respiration measured prior to ETC inhibitor addition. **B.** ATP-coupled respiration. **C**. Proton leak. **D.** Spare respiratory capacity. Data show the means of 3 biological replicates (A-D). **E.** Mitochondrion membrane potential (ΔΨm) was measured by detecting the incorporation of the TMRM in both regular growth medium and in restricted medium cultures. ΔΨm of *T*. *cruzi* strains were plotted relative to strain CL Brener. Data show the means of 4 biological replicates. **F.** Percent change in the mitochondrial membrane potential (ΔΔΨm) was calculated by subtracting the potential values in the restricted medium from the potential values in the exponentially growing cultures. Means of absolute values from 4 biological replicates were plotted. **G.** *T*. *cruzi* lysates of different strains from exponentially growing cells in regular growth medium were resolved by SDS-PAGE, transferred to PVDF membrane, and probed with *Leishmania major* COXIV (complex IV) antibody and *Trypanosoma brucei* β-subunit (ATP synthase) antibody. α-tubulin was used as a loading control. Representative blots are presented along with the normalized densitometry analysis of 4 biological replicates. Error bars represent the standard error of the mean. p-values shown below each plot were generated using one-way analysis of variance (ANOVA). *Post-hoc* Tukey’s test was performed to compare each data set to each other. * represents p ≤ 0.05 and ** represents p ≤ 0.01.

We are most interested in aspects of cellular function that involve the ETC because at least 15 of the 20 *T*. *cruzi* maxicircle genes encode ETC subunits [20], the expression of which we are analyzing in a separate study. *T*. *cruzi* ETC utilization is believed to be developmentally regulated [32,54,55], and it is possible that different strains may use the ETC or its components to different capacities. We examined ETC function indirectly by measurement of oxygen consumption in an extracellular flux-based stress assay. Oligomycin, FCCP, antimycin A and rotenone were used to sequentially inhibit the function of specific ETC complexes during the assay [33]. We scrutinized basal respiration, ATP coupled respiration, proton leak and spare respiratory capacity in each strain to identify differences between them (Fig 2). DNA quantitation per well with SYBR Green I dye fluorescence was used for normalization, as this value and cell number have a direct linear correlation as shown in S2 Fig. Although *T*. *cruzi* nuclear genome sizes vary by strain, both mitochondrial and nuclear genomes contribute to SYBR Green signal. Measurements show that differences in DNA content between CL Brener, Esmeraldo, and Tulahuen are negligible; the DNA content of Sylvio X10 will be less than these, but not likely to differ more than ~15% [16,17]. In fact, perusal of the raw DNA content output of all replicates in both medium conditions did not reveal a consistently lower or higher SYBR Green signal for any strain, suggesting that measuring total DNA content is an appropriate plating normalization control. S3 Fig shows complete normalized oxygen consumption rate profiles over the course of the experiments, performed in both normal and in restricted growth conditions.

Basal respiration, as the term is used here, is the parasites’ overall steady-state oxygen consumption, regardless of whether it is ATP-coupled. Strains have similar basal respiratory rates under both growth conditions with minor differences (Fig 2A). However, ATP-coupled respiration, the fraction of oxygen consumed that is coupled to ATP generation by ATP synthase, is significantly lower in Esmeraldo grown in normal medium compared with CL Brener and Tulahuen. The same trend is also observed in cells grown for 4 days in restricted medium. Similarly, Sylvio X10 appears to have a lower but not significantly different ATP-coupled respiratory rate compared to CL Brener and Tulahuen under both conditions (Fig 2B). The lack of basal oxygen consumption differences between CL Brener and Esmeraldo mirrored a previous comparison in these two cell lines; however, in that study differences in ATP-coupled respiration were not observed. The experiments were performed so differently that it is difficult to directly compare them [23]. Since it is unlikely that *T*. *cruzi* possesses glycosomal glycerol-3-phosphate dehydrogenase ([56,57] and also the genomic regions syntenic to the *T*. *brucei* protein are absent in all *T*. *cruzi* strains in TritrypDB), a *T*. *cruzi* G3P:DHAP shuttle is unlikely. Therefore, the mitochondrion is likely the sole contributor of ATP generation through the ETC, that is lower in Esmeraldo (and possibly Sylvio X10) than in the hybrid strains. If this is the case, substrate-level phosphorylation may play a larger role in energy generation in Esmeraldo and Sylvio X10, which could be the reason behind their higher medium acidification rates.

Interestingly, complex III activity in bloodstream trypomastigotes increases relative to that in epimastigotes, despite consistent complex IV activity levels between the life stages [55]. This results in the potential for higher rates of ROS formation, an effect that can be relieved by greater proton leak in that stage [58]. It is possible that efficiency of electron transport between complexes may be variable between strains. Therefore, we asked whether proton leak, quantified by mitochondrial consumption of oxygen that is not coupled to ATP generation, was enhanced in any strain. On the contrary, we found no significant strain variability in this parameter (Fig 2C), although we note the non-significant trend of Sylvio X10 showing a higher proton leak in regular growth conditions. It is possible that our assay is not sensitive enough to detect differences in this value that is normally fairly low.

Finally, we examined spare respiratory capacity (SRC), or the inherent maximal respiratory capacity minus the basal respiratory capacity, for each strain. Interestingly, strains Esmeraldo and Sylvio X10 have the lowest SRC, similar in magnitude to each other, and less than those of CL Brener and Tulahuen (Fig 2D). We interpret this to mean that Esmeraldo and Sylvio X10 have an inefficient ETC that they are utilizing at near-maximal capacity when glucose is both present and absent. To analyze efficiency further, we determined the fraction of ATP-coupled respiration relative to basal respiration rates. In normal medium, CL Brener and Tulahuen had fractions of 0.66 ±.09 and 0.61 ±.09, respectively, compared to relatively low fractions of 0.26 ±.12 for Esmeraldo and 0.34 ±0.19 for Sylvio X10. The difference was mirrored but less pronounced in restricted medium (CL Brener 0.59 ±.03, Tulahuen 0.64 ±0.06, Esmeraldo 0.42 ±0.10, Sylvio X10 0.30 ±0.3). These inefficiencies may force the lower efficiency strains to cope with changing energetic demands in ways that are different from more efficient strains: specifically, with an increased reliance on substrate-level phosphorylation, which would be consistent with their higher rates of medium acidification. The availability of CO_2_ in our growth conditions may have driven all strains to utilize the CO_2_-fixing glycosomal substrate level phosphorylation (resulting in succinate excretion) when glucose was present, which may have masked strain differences mitochondrial carboxylic acid output present in glucose-rich medium growth.

### Mitochondrial membrane potential (ΔΨm) conditionally differs among strains

The fact that differences exist in ETC use and capacity suggests that ΔΨm may also exhibit variability among these strains [59], which could in turn impact transport of ions, nutrients, and nuclear-encoded proteins across the mitochondrial membrane. For each strain, we determined mitochondrial membrane potential by measuring the fluorescent signal from tetramethylrhodamine (TMRM) that is sequestered by active mitochondria proportional to their ΔΨm. The ΔΨm does not differ among exponentially growing *T*. *cruzi* strains in the regular growth medium; however, there is strain variability in ΔΨm in the restricted medium at day 4 that contains no glucose (Fig 2E). Specifically, while ΔΨm drops in this condition for all cell lines relative to that of exponentially growing cells (Fig 2F), the magnitude of the decrease is strain-specific. In the restricted medium conditions, ΔΨm is significantly different in Sylvio X10 compared to Tulahuen, or to Esmeraldo, the strain in which ΔΨm changed the least relative to that of exponentially growing cells (Fig 2E and 2F). As these differences are only apparent under conditions of restricted growth, we also noted that in all ETC parameters tested, Sylvio X10 was the only strain potentially exhibiting differences between exponential and restricted growth, particularly in proton leak, although these differences did not reach the level of statistical significance. It appears that some aspect of growth in the absence of glucose interferes with strain Sylvio X10’s ability to maintain a consistent use of its ETC that in turn affects ΔΨm. For the other strains, differences in ETC use do not appear to be reflected in differences in ΔΨm.

### Limited evidence of strain-specific regulation of genes of energetic pathways exists

The lower respiratory capacity of Sylvio X10 and Esmeraldo relative to CL Brener and Tulahuen led us to ask if there were actually fewer ETC complexes in total in those strains. To test that hypothesis, we immunoblotted to detect nuclear-encoded subunits of complex IV and ATP synthase that are detectable in *T*. *cruzi* total protein extracts [12]. Irrespective of whether α-tubulin (Figs 2G and S4) or total protein (Coomassie stain, S4 Fig) was used for sample normalization, no strain variation in abundances of these subunits was observed. We presume this result to mean that there are similar numbers of core subunits of ETC complexes among strains and our hypothesis was incorrect. However, data from multiple proteomic studies revealed the presence of complex III and IV subunits even in the bloodstream form *T*. *brucei* where there is no evidence of fully assembled complexes or of their enzymatic activities [60]. Therefore, we cannot rule out that the relative abundances of the individual subunits that we tested are misleading.

Additionally, immunoblotting was limited to a single subunit of two of the five complexes comprising the ETC. It would fail to detect differences in ratios of subunits, particularly accessory proteins, or differences in subunit amino acid sequences that may result in different activities. For a wider perspective, we took advantage of high-throughput RNA sequencing read samples from CL Brener, Esmeraldo, and Sylvio X10 exponentially replicating epimastigotes that we have collected for mitochondrial transcriptome analysis. Because this purpose requires specialized sample preparation [38], reads may contain biases typically not found in high-throughput sequencing read populations. Nevertheless, samples (all harvested during exponential growth) from all strains were identically processed and can be used to identify strain variation in gene expression of classes of metabolic genes.

To our knowledge, only one other transcriptomic comparison of *T*. *cruzi* strains has been performed [61]. Except for a principle component analysis, this analysis did not directly compare overall degree of strain gene expression divergence, so we tested this first. Abundance of an mRNA within a transcriptomic dataset depends primarily on its stability and the copy number of the gene encoding that mRNA, as transcription is largely constitutive in trypanosomes. While *T*. *cruzi* genome size differences are largely attributed to insertions and deletions of tandem repeated sequence (repetitive elements) and telomeric repeats [16]; gene and chromosome copy number variations between strains are increasingly recognized [62]. The hybrid nature of CL Brener does not necessarily mean increases in gene copy numbers relative to the other two strains. However, since multiple laboratories have hypothesized that one of CL Brener parental lineages is thought to be the Esmeraldo-containing DTUII [40], we hypothesized that the transcriptomes of CL Brener and Esmeraldo would be more similar than that of Sylvio X10 and either of these strains.

**Fig 3:**
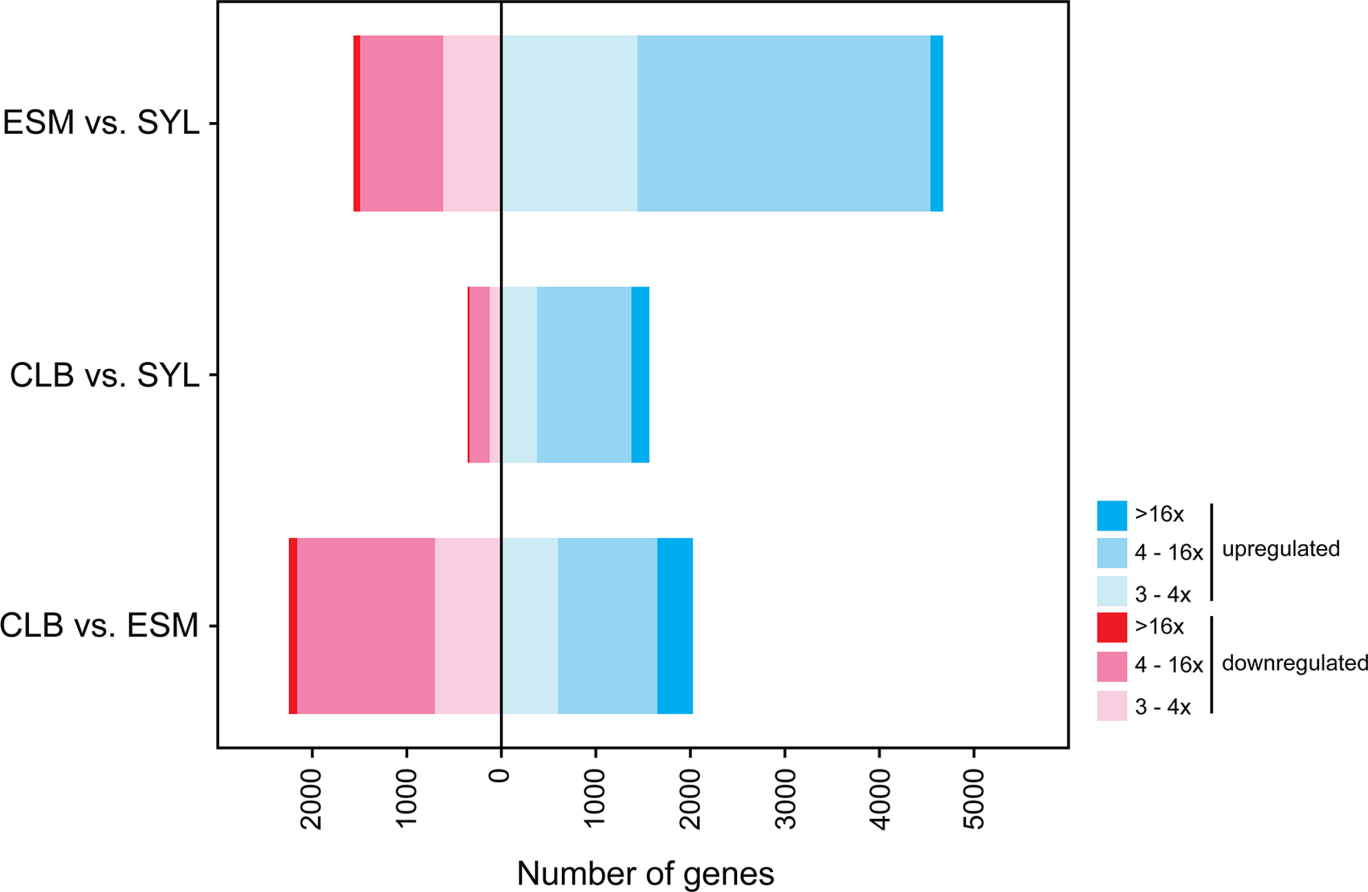
Differential gene expression between cultured *Trypanosoma cruzi* strains. Bar plots of the number of genes at least three-fold differentially expressed in each two-strain comparison listed on the y axis. ESM vs. SYL, expression in strain Esmeraldo relative to Sylvio X10; CLB vs. SYL, expression in strain CL Brener relative to Sylvio X10; CLB vs. ESM, expression in CL Brener relative to Esmeraldo.

Fig 3 conveys strain-to-strain comparisons of number and degree of at least three-fold differentially expressed genes. The x axis represents numbers of genes that are either negatively regulated (left; red) or positively regulated (right; blue) to a degree represented by color intensity. We observed the opposite of what we had hypothesized. The gene expression patterns of the smallest genome (Sylvio X10) and the hybrid genome (CL Brener) were relatively similar, while the expression patterns of the two non-hybrid strains (Sylvio X10 and Esmeraldo) had the greatest proportion of differentially expressed mRNAs. As a comparison, a similar analysis of CL Brener gene expression between trypomastigote and amastigote life stages [61] reveals at least as many genes are strongly differentially expressed (at least four-fold different) between strains as between life stages within a strain. Many of the most strongly differentially expressed genes between strains were surface proteins (mucins, mucin-associated surface proteins (MASPs), and trans-sialidases) that were part of multi-gene families, it is possible that the large-fold changes caused by copy-number variation. Similar percentages of uniquely mapped reads (60–65%) were present in datasets from all strains; evidence that reference bias potentially inherent to mapping all reads on the hybrid CL Brener reference is not a concern. This impression was confirmed with an additional analysis, the output of which can be found in S2 Table.

Particularly because of reports of major mutations in complex I subunits in some strains [20,22,23] we first looked for differential expression of mRNAs encoding ETC complex subunits identified in *T*. *brucei* or *T*. *cruzi* in proteomic studies [63–66]. We additionally examined the same metabolic pathways as Capewell et al. [67] in their *T*. *brucei* mammalian stage development study. These functional categories and the number of *T*. *cruzi* differentially expressed genes for strain pairwise comparisons within each category are shown in Table 1. All but one of the 138 ETC-associated *T*. *brucei* subunits had *T*. *cruzi* homologues, while 69% of the 307 *T*. *brucei* metabolic pathway genes had identifiable homologues in the CL Brener strain used as reference. Because of the acknowledged importance of translational control in *T*. *cruzi* differential gene expression [68], individual genes within metabolic pathways that are differentially expressed between strains may not be very relevant. Rather, we are looking for situations where multiple genes of a pathway are co-increased or decreased in abundance relative to other strains.

**Table 1:** Differential gene expression analysis of key metabolic pathways between *Trypanosoma cruzi* strains. The functional categories analyzed are listed in the first column; with the identities of genes within these categories provided in S3 Table. The total number of mRNAs that are at least three-fold differentially expressed between three pair-wise comparisons are shown in the next three columns. For the Esmeraldo vs. Sylvio X10 comparison, the percentage of analyzed *T*. *cruzi* genes that were found to be differentially expressed for each category is also shown. If the categories contained two or more differentially expressed Esmeraldo vs. Silvio X10 genes, the directionality of the regulation is presented (up or down). For regulation to be considered directional, zero or one gene only was permitted to be regulated in the opposite direction as the rest of the differentially expressed genes within that category. Strains analyzed are CL Brener (CLB); Sylvio X10 (Syl); Esmeraldo (Esm).

Functional Category	*T*. *brucei* genes	*T*. *cruzi* homologues	Differential regulation CLB vs. Esm	Differential regulation CLB vs. Syl	Differential regulation Esm vs. Syl		
					#	%	Directional
Complex I	48	47	3	1	11	23%	yes, up
Complex II	14	14	1	1	4	29%	no
Complex III	10	10	0	0	0	-	-
Complex IV	41	41	5	2	8	20%	no
ATP Synthase	22	22	2	1	6	27%	no
ETC Alternatives	3	3	0	0	0	-	-
Mitochondrial Carriers	26	18	1	0	2	11%	yes, up
Amino Acid Transport	32	24	7	0	7	29%	yes, up
TCA Cycle	17	9	1	0	1	11%	-
Acetate Metabolism	17	13	1	0	1	8%	-
NADH metabolism	4	2	0	0	1	50%	-
Lipid Metabolism	11	8	2	0	3	38%	yes, up
Glycolysis	46	33	4	3	11	33%	no
Pentose Phosphate Pathway	14	9	3	1	5	55%	no

Within examined gene categories as with the whole, transcriptomic expression was highly similar between CL Brener and Sylvio X10, with either zero or one mRNA per category being expressed at a level at least three-fold different between these strains. Exceptions were 3 of 33 glycolysis category enzymes being differentially expressed, and 2 of 41 complex IV components. Phenotypic differences between these two strains are therefore unlikely to be attributable to expression differences of the genes that we examined.

In contrast, strain Esmeraldo when compared to Sylvio X10 exhibited the greatest difference in mRNA abundances in the examined categories. In some categories such as amino acid transport, lipid metabolism, and complex I components, at least 23% of the genes were differentially expressed, and nearly all were upregulated in Esmeraldo. This suggests increased use of these pathways. Importantly we note that the increase in amino acid transporter mRNAs may result in some amino acids being more accessible as an energy source in Esmeraldo. Additionally, certain complex I subunits may have mutations in Esmeraldo that interfere with their function. It is possible that the increase in other subunits of this complex reflects a compensatory mechanism in this strain. In categories such as pentose phosphate pathway and glycolysis, and complexes II, IV, and ATP synthase, similar percentages of category genes were differentially expressed, yet genes were both up- and down-regulated in Esmeraldo relative to Sylvio X10. For the ETC complexes, most up- or down-regulated proteins are not enzymatic components of the complex. It is thus possible that the products of the differentially expressed genes may be more or less part of these complexes. This could potentially affect complex activity without a change in abundances of their core components. However, neither our transcriptomic nor our protein immunoblot analysis indicates a global up- or down-regulation of the entire ETC in any strain.

### Strains have distinct mitochondrion morphologies

**Fig 4:**
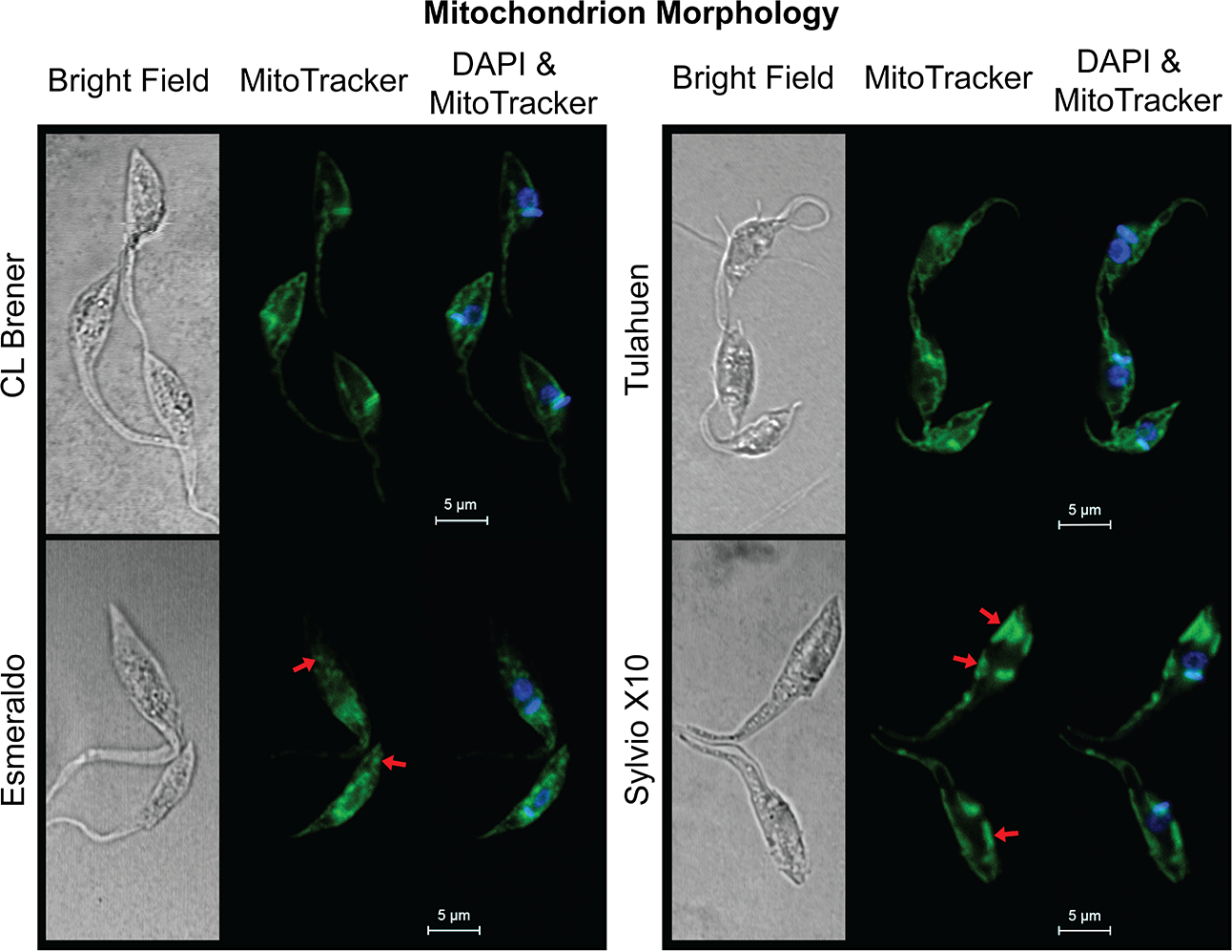
Different strains have unique mitochondrion morphologies. Mitochondrion morphologies of four *Trypanosoma cruzi* strains were determined using MitoTracker Deep Far Red FM probe (shown in green for easier visualization). Exponentially growing parasites in normal medium (LIT) were stained with 250 nM MitoTracker in PBS, fixed with paraformaldehyde and counter stained with DAPI following fixation. Cells were visualized and qualitatively described for three biological replicates. Representative images for each strain are presented. Arrows in Esmeraldo point to punctate mitochondrial structures, and arrows in Sylvio X10 indicate larger mitochondrial blobs positioned close to the cell membrane.

Since we observed strain variability in both ETC function and ΔΨm, we examined mitochondrion morphology to identify any strain-specific gross structural differences in the mitochondrion. In a qualitative but reproducible analysis, we observed that mitochondria of different strains exhibited unique morphologies (Fig 4). Mitochondria of strains CL Brener and Tulahuen displayed an especially web-like pattern of extensive reticulation and were nearly indistinguishable from one another. In contrast, the vesicular web of the Esmeraldo mitochondrion exhibited a more punctate appearance, like stars in a night sky (arrows, Fig 4 bottom left panel), while the single mitochondrion of Sylvio X10 appeared to be a network of irregularly grouped larger clumps of material predominantly located near the cell membrane (arrows, Fig 4 bottom right panel). These patterns were consistent whether we were examining cells from normal growth conditions shown in Fig 4 (additional images in S5 Fig), or restricted growth conditions.

We attempted to align these results with the strain-specific variability of ETC function and ΔΨm. We noted that the strain that is most clearly the outlier in mitochondrial morphology is Sylvio X10, and this strain also exhibits low ATP-coupled respiration and spare respiratory capacity, and the lowest ΔΨm in a restricted growth condition. It is possible that the less evenly distributed nature of the Sylvio X10 mitochondrion is responsible for the other differences seen in this strain relative to the others examined, or vice-versa. There are also a multitude of potential sources of morphological variation beyond the functional categories that we have investigated, including those which impact membrane protein and lipid compositions [69,70], or mitophagy. While electron microscopy ultrastructural studies would be necessary to truly define the differences in structure, it is clear that mitochondrial morphology exhibits phenotypic variation between strains.

## Discussion

This study’s focus was identification of strain differences in metabolic processes that could be impacted by differences in both the nuclear and the mitochondrial genome, in strains frequently used for molecular studies of cultured *T*. *cruzi*. We did not find a cohesive set of phenotypes for any strain that would definitively link its unique profile to a specific metabolic pathway or pathways. Other studies have likewise encountered such complexity. A study in which various nuclear-encoded subunits of ETC complex IV were silenced in *T*. *brucei* found that the severity of ATP production, growth, and ΔΨm phenotypes among the silenced lines was not consistent and yielded few patterns on which to base a model [71]. Another study focused on the impact of *T*. *cruzi* complex I mutations were unable to link response to H_2_O_2_ and ROS generation [23]. The interdependencies of trypanosome metabolic pathways are not yet well established, particularly for *T*. *cruzi*. However, we have been able to establish a few tentative links between phenotypes in strains on which to base future investigations.

Surprisingly, even strains in the same DTU, Tulahuen and CL Brener, exhibited differences in growth (Fig 1), although in tested characteristics of mitochondrial form and function (Figs 2 and 4), they exhibited near-identical phenotypes. Esmeraldo was the slowest growing strain in normal medium and exhibited the greatest medium acidification rates when they were tested in a more restrictive culture growth environment (Fig 1). Esmeraldo’s ATP-coupled respiration rate was significantly lower than that of the CL Brener and Tulahuen strains (Fig 2B), which could be linked to its lower maximal respiratory capacity (Fig 2D). One potential cause of the reduced capacity of the Esmeraldo ETC are the known mutations in mitochondrially-encoded subunits of complex I [20,23]. However, studies in various trypanosomes have questioned whether a substantial quantity of electrons really enter the trypanosome ETC at complex I [72]; most are thought to be transferred from succinate at complex II. Whatever the cause of the reduced respiratory capacity for this strain, it is not coupled with a relatively lower ΔΨm (Fig 2E), and the overall mitochondrial morphology of the Esmeraldo strain is only subtly altered from that of CL Brener and Tulahuen.

Despite being most dissimilar in relative gene expression levels (Fig 3 and Table 1), the Esmeraldo and Sylvio X10 strains have metabolic similarities. They both acidify medium faster than the DTU VI strains (Fig 1D), a fact possibly related to their curtailed use of the ETC relative to substrate-level phosphorylation to generate ATP (Fig 2B). But for Sylvio X10, potential deficiencies in spare respiratory capacity and ATP-coupled respiration are paired with an relatively smaller ΔΨm particularly when utilizing amino acids as an energy source (Fig 2E). Possibly related to this difference in membrane potential is a rather distinct mitochondrial morphology (Fig 4). Therefore, even though Sylvio X10 and Esmeraldo have similar oxygen consumption profiles, the causes for reduced maximal capacity may be very different. As a consequence, the two strains may experience differing amounts of oxidative stress, caused by differing amounts of mitochondrially-produced ROS. This could have long-ranging consequences. For example, even with no glucose in the medium, *T*. *cruzi* may still require use of the pentose phosphate pathway to generate NADPH in order to cope with oxidative stress. Therefore, variable levels of gluconeogenesis could occur with byproducts of mitochondrial metabolism that are exported out of the mitochondrion (such as malate; see diagrams in [53]), to feed the pentose phosphate pathway. In fact, in a recent study, low amounts of intracellular glucose were found in cultured epimastigotes long after it was gone from the medium [31].

Discovering connections between strain genetic and metabolic phenotypic difference is the long-term goal of this work. Strain specific differences of even single amino acids in ETC subunits can profoundly affect function and fitness in other organisms [73,74]. The extent of strain genetic diversity within *T*. *cruzi* [15,75] makes this a probable explanation for strain-specific metabolic differences where the origins were clearly not at the level of differential protein or enzyme expression (Table 1). Although we determined that our inter-strain transcriptomic analysis was not substantially impacted by the fact that one of the strains was a hybrid and two were not, a hybrid genome can influence phenotype in other ways. Most obviously, in our study it would provide CL Brener and Tulahuen strains a layer of genetic flexibility lacking in Sylvio X10 and Esmeraldo that can be advantageous over time. Relatedly, the inherent flexibility that these parasites possess that allows them to adapt to various environments can present a scientific challenge when integrating strain-specific information from multiple studies. Briefly, clones or even original cultures over time can change their genetic character (in a variation of clonal expansion) substantially enough to respond differently to identical environmental stimuli. For instance, an early experiment has already demonstrated that multiple clones of a non-clonal *T*. *cruzi* strain excrete different proportions of a variety of metabolites into culture medium, proportions that can also differ from that of the parent strain [76]. Indeed, we cannot rule out that some form of clonal expansion of strains in our hands has influenced the results that we present here.

Membrane potential and ETC function variability may be the result of differences in mitochondrially-encoded proteins that are part of the ETC, as well as nuclear genome-derived variability. Expression of mitochondrially-encoded functional mRNAs, and even the sequence of the expressed protein could potentially be impacted by mitochondrial RNA editing. While not proven at the protein level, recent high-throughput sequencing studies have shown different mitochondrially-encoded mRNAs to be generated from transcripts of a single mitochondrial genetic locus through editing [38,77,78]. Overall abundances of correctly edited and thus translatable mRNAs, or the balance of various potentially translated alternative mRNAs, could vary among strains, and is the focus of our other study that is currently underway.

The cause of differential medium acidification between strains, observed after the cells can no longer rely on culture glucose for energy, is yet to be resolved. pH decreases of culture medium likely come about due to excreted carboxylic acid metabolites. It is not expected that appreciable amounts of lactic acid, the metabolite mainly responsible for mammalian cell culture acidification would be excreted by *T*. *cruzi* [48]. However, succinate, acetate, and other metabolites are excreted in variable quantities in *T*. *cruzi* amino acid catabolism [79,80]. For example, work by Barisón et al. [81] shows that histidine can be completely metabolized to CO_2_ by epimastigotes, and variable utilization of this and other amino acid catabolic pathways may result in differing amounts of CO_2_ relative to carboxylic acid release. Since metabolic intermediates can either be directly excreted upon mitochondrial substrate-level phosphorylation, donate some electrons to oxidative phosphorylation, or even be transferred to the cytosol or glycosome to feed anabolic and catabolic pathways that reside there, it is probably necessary to utilize metabolomic approaches [82] to determine whether and how excreted metabolic byproducts differ among strains.

We also noted variability in individual replicates in our mitochondrial stress assay. Therefore, we have possibly missed all but the most extreme strain-specific differences in utilizing this method. Finally, our study focused on a subset of strains that are all available from a reliable repository (ATCC) and for which mitochondrial as well as nuclear genomes have been sequenced. Strains from DTUs, III, IV, and V that were not analyzed here may exhibit further variation. We conclude that metabolic *T*. *cruzi* strain variability, of even the limited pathways that we analyzed here, is extensive enough that the use of more than strain should be considered in future culture molecular studies with these sequenced strains. These differences present opportunities as well as challenges. The identified phenotypic variabilities and differences in gene expression suggest fruitful avenues for future investigation. Importantly, these avenues of in vitro study may help to identify the metabolic pathways that most influence replication, differentiation, and transmission, in order to inform necessary but challenging in vivo studies.

## Supporting information

S1 Table *Trypanosoma brucei* genes included in each functional category used in differential gene expression analysis. The numbers of genes in each category with functional *Trypanosoma cruzi* homologues are provided in Table 1.
(XLSX)

S2 Table Three potential references were tested for mapping RNA-seq data using the parameters described in materials and methods. CL Brener is a hybrid genome. One set of its chromosomes encode genes described as “Esmeraldo-like” because the parental lineage of that genome is DTU II (as is the non-hybrid strain Esmeraldo). The other set is described as “non-Esmeraldo-like” and has a different parental lineage. One of the three references we tested was the diploid CL Brener genome reference from El-Sayed *et al*. (10.1126/science.1112631), named "Hybrid" in this table. Also tested were the separate Esmeraldo-like (ESM) and Non-Esmeraldo-like (Non-ESM) haplotype genomes available at TriTrypDB. We mapped the RNA-seq datasets for CL Brener and Esmeraldo onto each of these references. Results are summarized, showing reads mapped on each reference and the portion of mapped reads that were captured between all possible pairings of references. Nearly all CL Brener reads mapped to either haplotype also mapped to the diploid hybrid, as did the Esmeraldo reads of this non-hybrid strain, with no appreciable differences in mapping performance for each strain’s RNA-seq dataset. The Hybrid reference genome provides the most complete mapped read set for all genomes and thus was the one selected.
(XLSX)

S3 Table *Trypanosoma cruzi* genes that were three-fold differentially expressed in three pair-wise comparisons between strains. (XLSX)

S1 Fig Strains respond variably to in vitro life cycle differentiation protocol. **A.** *Trypanosoma cruzi* grown for 8 days in restricted medium (RPMI and 1% FBS) as previously performed were collected at day 8, then fixed in paraformaldehyde and stained with DAPI for fluorescence microscopy [12]. At least 100 cells per sample were analyzed for overall morphology and organellar positioning. Different life stages were identified as follows: Parasites with anteriorly located kinetoplast—epimastigotes; posteriorly located kinetoplast—trypomastigotes; and possessing typical trypomastigote cell morphology with a kinetoplast positioned at the center of the nucleus or beyond but was not fully posterior—intermediates. Parasites with round and indistinct anterior/posterior morphologies were identified as spheromastigotes. Stacked bars show the mean percentages of each life stage from 3 biological replicates. Error bars represent the standard error of the mean (SEM). **B.** To confirm results shown in (A), the same cells collected at day 8 along with epimastigotes collected from exponentially growing cultures (D0 LIT) were analyzed for survival when exposed to active guinea pig serum. Epimastigotes are lysed in the presence of serum under the conditions used [12]; the cells resistant to serum represent the trypomastigotes of the mixed culture. Sylvio X10 epimastigotes as well as trypomastigotes are largely serum-resistant, so this strain was not included. Stacked bars show the mean percentages of lysed cells relative to identical treatment with heat-inactivated serum (HI) from 2 technical replicates of a single biological replicate. Error bars represent the SEM.
(PDF)

S2 Fig Linear regression analysis of the relationship between cell number and the SYBR Green 1 fluorescence. *Trypanosoma cruzi* were serially diluted and seeded in a 96-well SeaHorse (Agilent) assay plate. Medium was gently removed and cells were lysed in the lysis buffer overnight. Fluorescence signals per well were detected for the range of cells/well shown. Linear regression analysis resulted in an R-square value of 0.916, demonstrating good correlation between SYBR Green 1 fluorescence and cell number. Each data point represents the means of 4 technical replicates. Dotted lines delineate the 95% confidence interval for the regression curve. Error bars represent the SEM.
(PDF)

S3 Fig Normalized respiratory profiles for four *Trypanosoma cruzi* strains. Mitochondrial stress test extracellular flux assays were performed on *T*. *cruzi* epimastigotes that had been grown in culture conditions for normal (left panel) and restricted (right panel) growth. The y axis indicates normalized oxygen consumption rates (OCR) over time (x axis). Measurements were taken at indicated time points. The following drugs were introduced at time points shown by dashed vertical lines: Oligo, oligomycin; FCCP, trifluoromethoxy carbonylcyanide phenylhydrazone; AA/Rot, antimycin A and rotenone. Error bars represent the SEM.
(PDF)

S4 Fig Protein abundances of nuclear encoded ETC subunits among stains in regular and restricted growth medium along with loading controls. *Trypanosoma cruzi* lysates of different strains from exponentially growing cells in regular growth medium and from slow growing cells harvested after 4 days growth in restricted medium were resolved by SDS-PAGE, transferred to PVDF membrane, and probed with *Leishmania major* COXIV (complex IV) antibody and *Trypanosoma brucei* β-subunit (ATP synthase) antibody. α-tubulin was used as a loading control. Representative blots are presented along with the normalized densitometry analysis of 4 biological replicates. Error bars represent the standard error of the mean. p-values were calculated by using ordinary one-way analysis of variance to assess the significance of the observed minor differences in abundance and are shown below each densitometry bar graph. **Coomassie stains of gels equally loaded with the same samples are shown as a loading control alternative.** Panels of representative blots and quantitative analysis for exponential cells as those presented in Fig 2G.
(PDF)

S5 Fig Additional images highlighting unique mitochondrion morphologies of four *Trypanosoma cruzi* strains. Exponentially growing parasites in normal medium (LIT) were stained with 250 nM MitoTracker Deep Far Red FM probe in PBS, fixed with paraformaldehyde and counter stained with DAPI following fixation. Regions of the Esmeraldo mitochondrion present with a punctate “starry night” appearance, relative to the more clearly networked CL Brener and Tulahuen mitochondria, or the Sylvio X10 larger networked mitochondrial blobs positioned close to the cell membrane.
(PDF)
