# Supplementary material for: Gene expression to mitochondrial metabolism: Variability among cultured Trypanosoma cruzi strains
Source: PLoS One. 2018 May 30;13(5):e0197983. doi: 10.1371/journal.pone.0197983 (PMC5976161; doi:10.1371/journal.pone.0197983)

Supplemental Figure 1.
(Related to Figure 1)

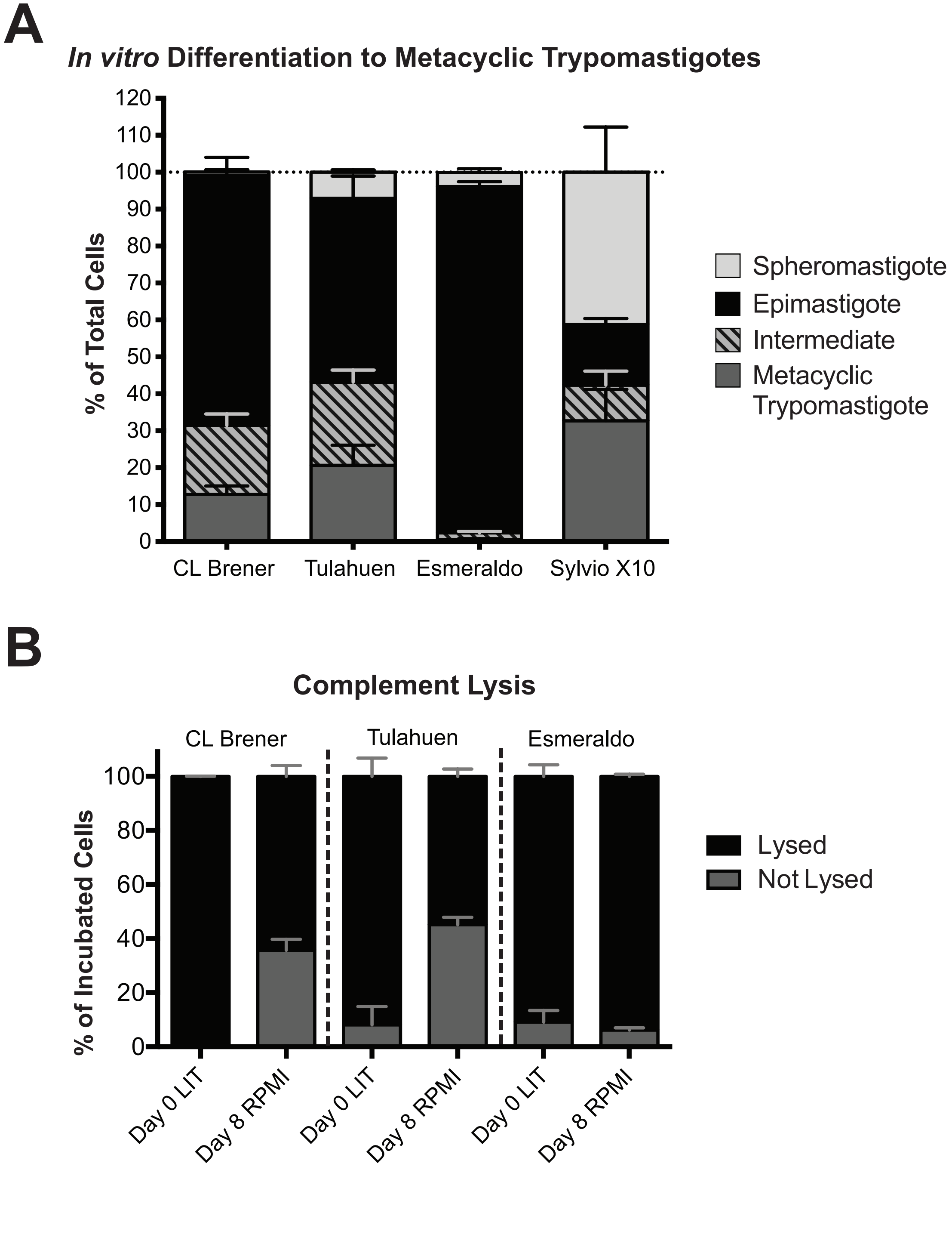

Supplement: S1 Fig — A. Trypanosoma cruzi grown for 8 days in restricted medium (RPMI and 1% FBS) as previously performed were collected at day 8, then fixed in paraformaldehyde and stained with DAPI for fluorescence microscopy [12]. At least 100 cells per sample were analyzed for overall morphology and organellar positioning. Different life stages were identified as follows: Parasites with anteriorly located kinetoplast—epimastigotes; posteriorly located kinetoplast—trypomastigotes; and possessing typical trypomastigote cell morphology with a kinetoplast positioned at the center of the nucleus or beyond but was not fully posterior—intermediates. Parasites with round and indistinct anterior/posterior morphologies were identified as spheromastigotes. Stacked bars show the mean percentages of each life stage from 3 biological replicates. Error bars represent the standard error of the mean (SEM). B. To confirm results shown in (A), the same cells collected at day 8 along with epimastigotes collected from exponentially growing cultures (D0 LIT) were analyzed for survival when exposed to active guinea pig serum. Epimastigotes are lysed in the presence of serum under the conditions used [12]; the cells resistant to serum represent the trypomastigotes of the mixed culture. Sylvio X10 epimastigotes as well as trypomastigotes are largely serum-resistant, so this strain was not included. Stacked bars show the mean percentages of lysed cells relative to identical treatment with heat-inactivated serum (HI) from 2 technical replicates of a single biological replicate. Error bars represent the SEM. (PDF) [file pone.0197983.s004.pdf]
