# Supplementary figures and images for: Gene expression to mitochondrial metabolism: Variability among cultured Trypanosoma cruzi strains
Source: PLoS One. 2018 May 30;13(5):e0197983. doi: 10.1371/journal.pone.0197983 (PMC5976161; doi:10.1371/journal.pone.0197983)

# Supplemental Figure 2.

(Related to Figure 2)

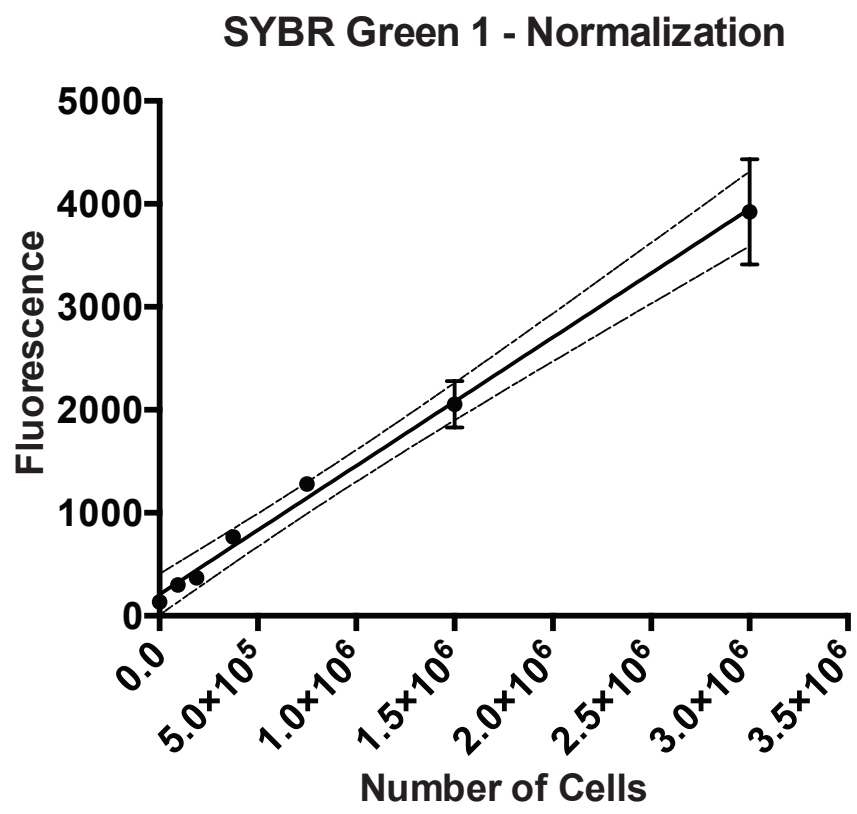

Supplement: S2 Fig — Trypanosoma cruzi were serially diluted and seeded in a 96-well SeaHorse (Agilent) assay plate. Medium was gently removed and cells were lysed in the lysis buffer overnight. Fluorescence signals per well were detected for the range of cells/well shown. Linear regression analysis resulted in an R-square value of 0.916, demonstrating good correlation between SYBR Green 1 fluorescence and cell number. Each data point represents the means of 4 technical replicates. Dotted lines delineate the 95% confidence interval for the regression curve. Error bars represent the SEM. (PDF) [file pone.0197983.s005.pdf]

# Supplemental Figure 3.

(Related to Figure 2)

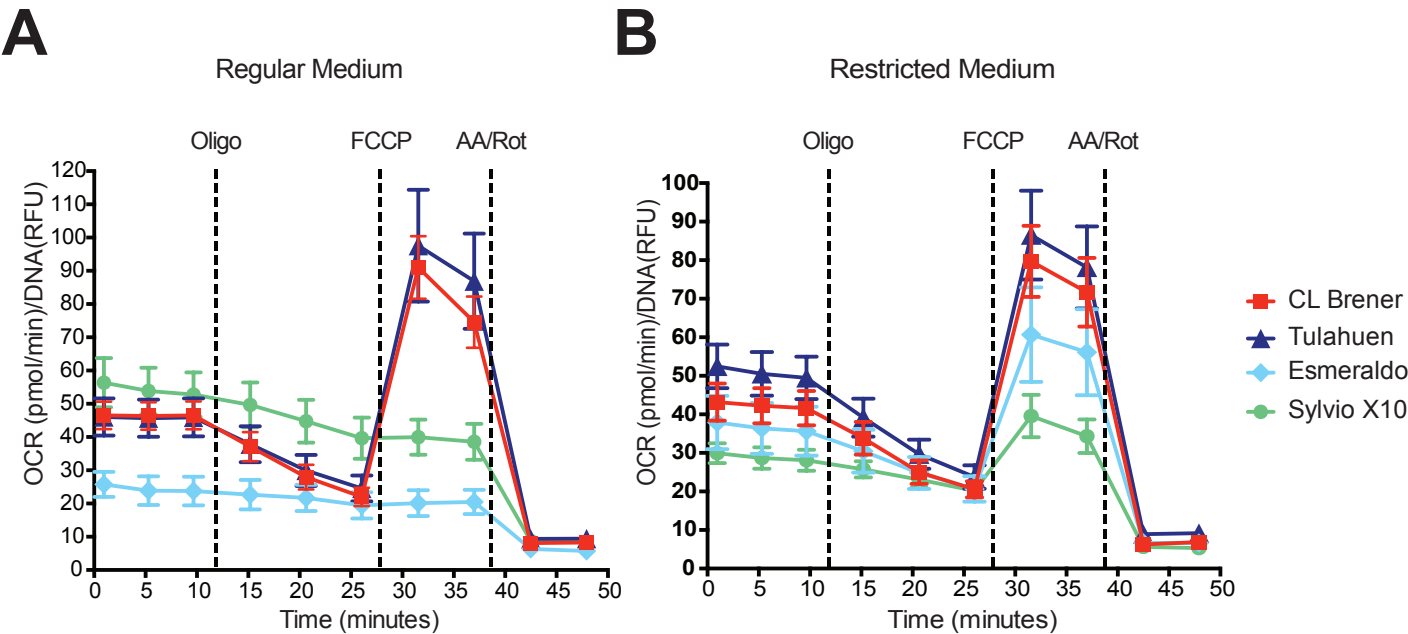

Supplement: S3 Fig — Mitochondrial stress test extracellular flux assays were performed on T. cruzi epimastigotes that had been grown in culture conditions for normal (left panel) and restricted (right panel) growth. The y axis indicates normalized oxygen consumption rates (OCR) over time (x axis). Measurements were taken at indicated time points. The following drugs were introduced at time points shown by dashed vertical lines: Oligo, oligomycin; FCCP, trifluoromethoxy carbonylcyanide phenylhydrazone; AA/Rot, antimycin A and rotenone. Error bars represent the SEM. (PDF) [file pone.0197983.s006.pdf]
