# Supplementary material for: Gene expression to mitochondrial metabolism: Variability among cultured Trypanosoma cruzi strains
Source: PLoS One. 2018 May 30;13(5):e0197983. doi: 10.1371/journal.pone.0197983 (PMC5976161; doi:10.1371/journal.pone.0197983)

Supplemental Figure 4.
(Related to Figure 2)

Immunoblotting: Nuclear-encoded ETC subunits

ß-subunit - Complex 5

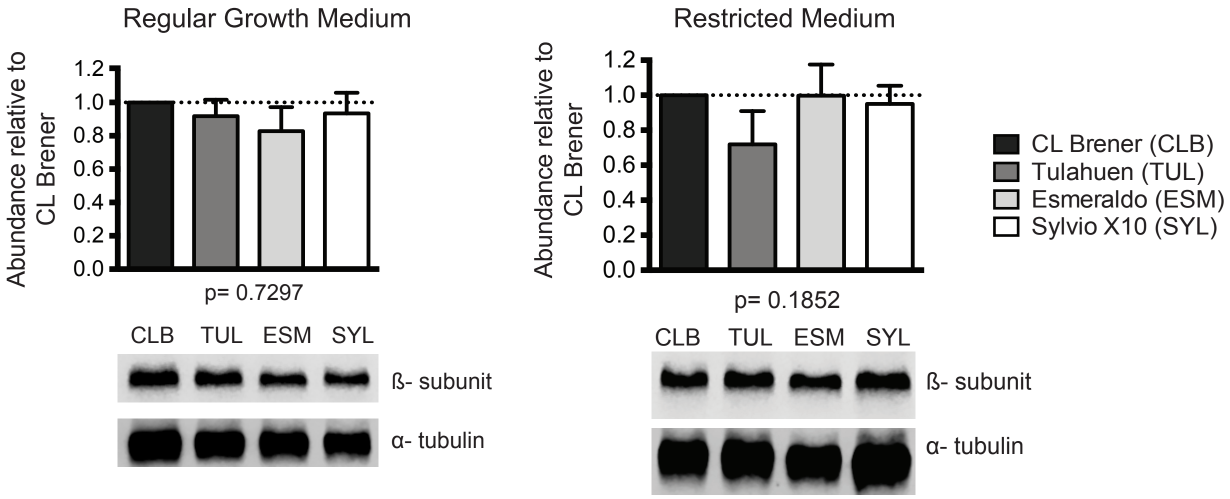

COX IV - Complex 4

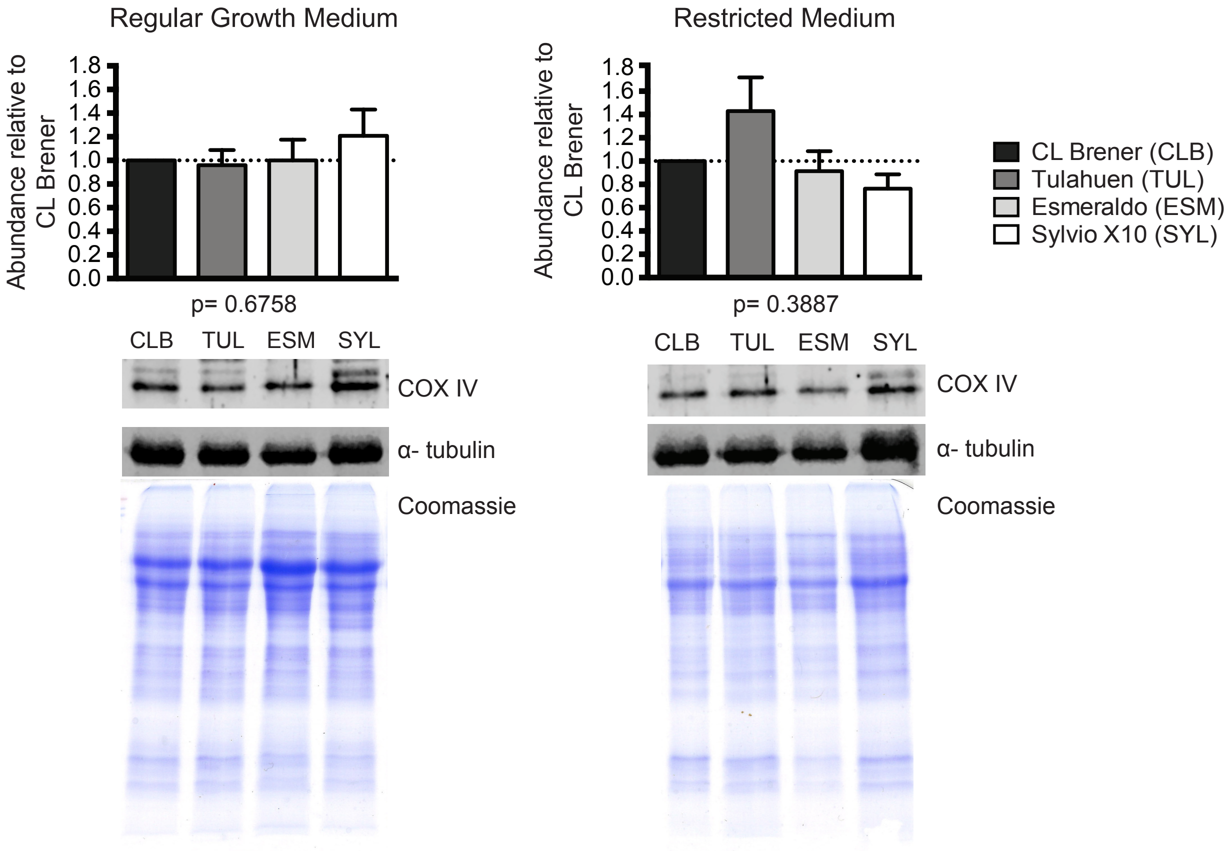

Supplement: S4 Fig — Trypanosoma cruzi lysates of different strains from exponentially growing cells in regular growth medium and from slow growing cells harvested after 4 days growth in restricted medium were resolved by SDS-PAGE, transferred to PVDF membrane, and probed with Leishmania major COXIV (complex IV) antibody and Trypanosoma brucei β-subunit (ATP synthase) antibody. α-tubulin was used as a loading control. Representative blots are presented along with the normalized densitometry analysis of 4 biological replicates. Error bars represent the standard error of the mean. p-values were calculated by using ordinary one-way analysis of variance to assess the significance of the observed minor differences in abundance and are shown below each densitometry bar graph. Coomassie stains of gels equally loaded with the same samples are shown as a loading control alternative. Panels of representative blots and quantitative analysis for exponential cells as those presented in Fig 2G. (PDF) [file pone.0197983.s007.pdf]
