# Supplementary material for: Gene expression to mitochondrial metabolism: Variability among cultured Trypanosoma cruzi strains
Source: PLoS One. 2018 May 30;13(5):e0197983. doi: 10.1371/journal.pone.0197983 (PMC5976161; doi:10.1371/journal.pone.0197983)

Supplemental Figure 5.
(Related to Figure 4)

Mitochondrion Morphology in Regular Growth Medium, LIT.

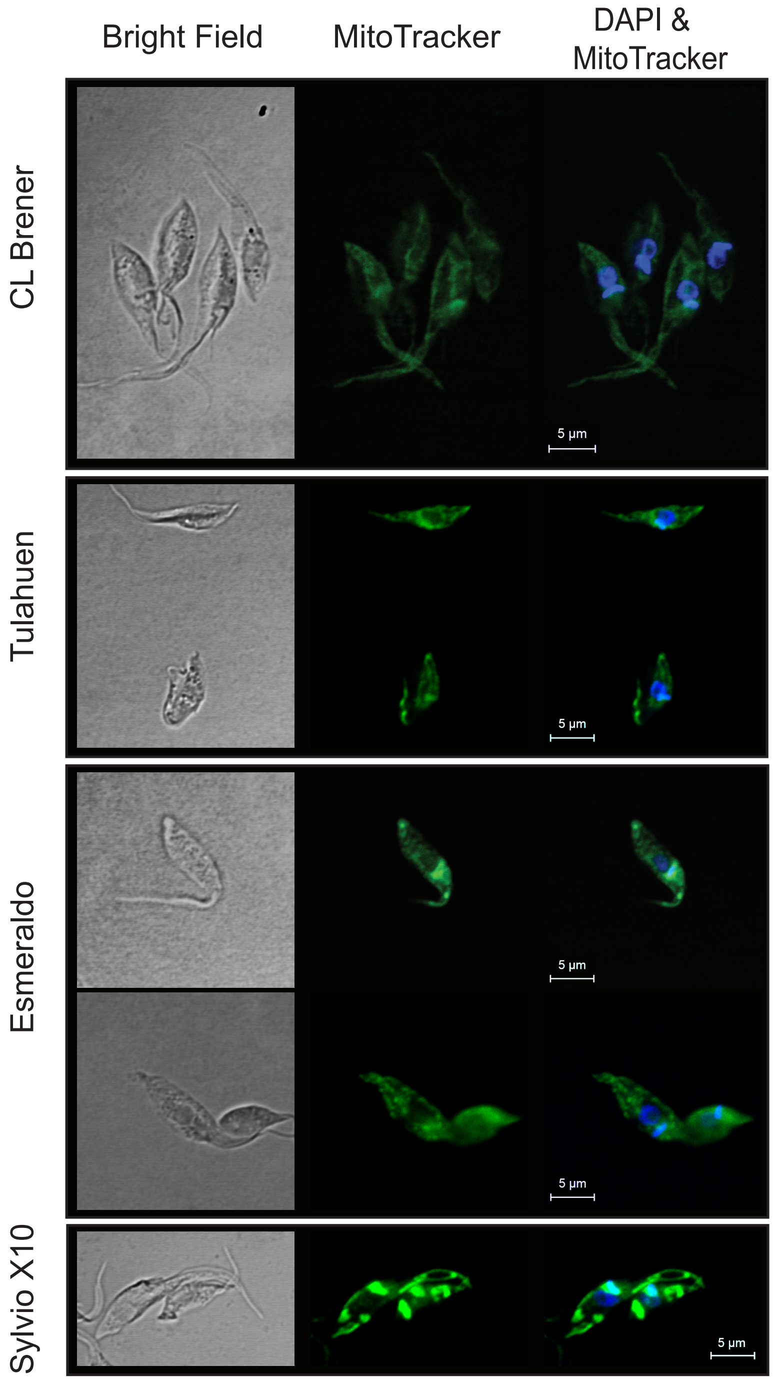

Supplement: S5 Fig — Exponentially growing parasites in normal medium (LIT) were stained with 250 nM MitoTracker Deep Far Red FM probe in PBS, fixed with paraformaldehyde and counter stained with DAPI following fixation. Regions of the Esmeraldo mitochondrion present with a punctate “starry night” appearance, relative to the more clearly networked CL Brener and Tulahuen mitochondria, or the Sylvio X10 larger networked mitochondrial blobs positioned close to the cell membrane. (PDF) [file pone.0197983.s008.pdf]
